# Insights into the Pathophysiology of Post-COVID Lung Disease from the Perspective of Histopathology

**DOI:** 10.1159/pat/aehag004

**Published:** 2026-10-10

**Authors:** Thomas Menter, Kirsten D. Mertz, Alexandar Tzankov

**Affiliations:** Pathology, Institute of Medical Genetics and Pathology, University Hospital Basel, Basel, Switzerland

**Keywords:** SARS-CoV-2, Angiotensin-converting enzyme 2, Neuropilin, Endothelial cell, Lung fibrosis

## Abstract

**Background:**

The COVID-19 pandemic has caused a broad range of respiratory complications. Among the spectrum of COVID-associated pulmonary conditions, severe bilateral pneumonitis that might progress to acute respiratory distress syndrome represents the most critical manifestation. A substantial proportion of survivors of severe COVID-19 continue to experience persistent respiratory symptoms and functional limitations. These findings are referred to as post-acute sequelae of COVID-19.

**Summary:**

These residual post-COVID lung abnormalities are mechanistically linked to the structural changes of acute lung injury, but also to persistent systemic inflammatory signatures, and are reflected in a range of histopathological patterns, including pulmonary fibrosis, hyperinflammation, angiopathic and immunothrombotic tissue responses.

**Key Messages:**

This review focuses on the current knowledge of the pathogenesis and pathophysiology of post-COVID lung disease. We also schematically delineate the broad spectrum of post-COVID lung disease and explain the underlying pathomechanisms.

## Introduction

The COVID-19 pandemic has brought unprecedented challenges to healthcare systems worldwide, emphasizing the profound effects of pandemics on a globalized population [1]. The emergence of associated post-acute sequelae of COVID-19 (PASC), also referred to as long COVID, has become a new global health crisis secondary to infection [2]. PASC is a systemic syndrome but, given the proclivity of SARS-CoV-2 for inducing lung injury, there is a large burden of long-term pulmonary manifestations that merit specific attention. In survivors of severe COVID-19, residual respiratory sequelae may span from symptoms and functional limitations not associated with clear-cut structural correlates to debilitating post-COVID interstitial lung disease (post-COVID ILD) that may considerably compromise respiratory function and quality of life. This wide range of pulmonary manifestations is a global public health challenge, affecting millions worldwide [3].

Post-COVID lung impairment includes a diverse range of dysfunctions that persist long after the initial acute infection has resolved. Its diagnosis relies on the identification of persistent functional and/or radiological lung abnormalities in individuals who have recovered from COVID-19. The healthcare implications are substantial, with an increased demand for long-term medical management and recurrent hospitalizations, culminating in the need for lung transplant in some patients. The economic burden is equally substantial, as these conditions lead to considerable healthcare expenditures and productivity losses due to prolonged disability [3]. Beyond the medical and economic impact, post-COVID lung injury often leads to drastic lifestyle changes and is often accompanied by psychological distress, with many individuals suffering from anxiety or depression [4].

This review focuses on the current understanding of the pathogenesis and pathophysiology of post-COVID lung disease. The basis for this review includes the results of published and unpublished data of our research group as well as evidence from PubMed searches on the various topics of this review using the keywords “histopathology,” and “long COVID,” “post COVID,” “persistent COVID,” “post-acute sequelae COVID,” “chronic sequelae COVID,” “late sequelae COVID,” “lung fibrosis COVID,” or “end stage COVID.”

## Epidemiology and Prevalence

As of March 2026, there have been more than 779 million reported COVID-19 cases and 7 million deaths worldwide [5]. Due to incomplete reporting, lack of tracking, and limited surveillance in many countries, these figures underestimate the true global impact of SARS-CoV-2. As severity of the acute illness is a key driver of post-COVID-19 lung disease, it is important to understand the temporal context and clinical setting of the initial infection when discussing the long-term sequelae [3].

The global burden of acute COVID-19 has changed substantially over time due to the emergence of new viral variants, widespread access to vaccination, and more evidence-based treatment strategies. Transmissibility, virulence, and pathogenicity of different SARS-CoV-2 variants, together with availability of local healthcare resources and stress on healthcare systems, contribute to the heterogeneity in disease burden and severity observed across populations. The availability of effective vaccines against SARS-CoV-2 in late 2020 dramatically decreased the burden of severe acute illness and has also been shown to reduce the risk of post-COVID respiratory symptoms and chronic lung disease [6], as the virus lost its dependence on transmembrane protease serine subtype 2 [7], and by means of this, its pulmotropism while infecting much more efficiently the upper respiratory tract (nasal epithelium), thus dramatically decreasing lung injury.

Given the wide spectrum of manifestations of both acute infection and post-COVID lung disease, incidence and prevalence estimations vary widely depending on the population studied and the time point after infection. This is further complicated by the lack of standardized and validated parameters to objectively quantify disease burden. A meta-analysis including 33 studies reported a global prevalence of post-COVID lung disease of 43% [8], with rates of 54% among hospitalized and 34% among nonhospitalized patients. Prevalence rates are lower (13.6%) when electronic health records are used for outcome ascertainment [9]. Dyspnea is the most reported respiratory symptom, observed in 10–77% of individuals who recover from acute COVID-19 [10]. The underlying mechanisms of dyspnea in this population are heterogenous, as many patients have no abnormalities on chest computed tomography (CT) scans or pulmonary function tests. Cough is reported in 17–70% of patients during the recovery phase, persisting for up to 1 year [11, 12]. The occurrence of interstitial lung disease (ILD), or lung fibrosis, following acute viral infections is not a new phenomenon and has been reported in outbreaks of H1N1, SARS, and MERS-CoV. ILD develops in only a minority of patients after acute SARS-CoV-2 infection [13] and is rarely progressive, except in patients with preexisting ILD or interstitial lung abnormalities. In general, post-COVID-19 lung injury is subdivided into three categories (Table 1): (1) organizing diffuse alveolar damage (DAD) as the histopathological pattern of acute respiratory distress syndrome (ARDS), which may end in progressive fibrosis, (2) post-COVID-19 pulmonary fibrosis strongly resembling nonspecific interstitial pneumonia (NSIP) and linked to vascular remodeling characterized by prominent intussusceptive neoangiogenesis and distinct macrophage dysfunction, and (3) virus-induced exacerbation of preexisting subclinical ILD, with various histological patterns.

**Table 1.** Differential pathobiology of post-COVID lung patterns ([14–19] and own observations)

Pattern	Trigger	Morphology	Outcome
Organizing DAD	Acute ARDS	See Table 3	Gradual resolution
Post-COVID pulmonary fibrosis	Vascular and/or immune compound dysfunction		Persistent and debilitating, but stable
Exacerbation of preexisting ILD	Second hit on the background of clinical/subclinical ILD		High risk of rapid progression

ARDS, acute respiratory distress syndrome, COVID, coronavirus disease, DAD, diffuse alveolar damage, ILD, interstitial lung disease.

In the last years, many new insights into the genetic pathogenesis of idiopathic pulmonary fibrosis (IPF) have been gained, especially in cases with a potential family history [20]. These mutations comprise telomere-related and surfactant-related genes as well as susceptibility loci such as the *MUC5B* minor T-allele. Thus, the genetic background might also provide a helpful clue at least in a subset of patients. Yet it has to be kept in mind that in cases without a strong family history of IPF, well-characterized mutations are only present in 5–10% of cases, and *MUC5B* variant alleles also have a relative high prevalence in healthy controls (40% in IPF patients versus 10% in healthy controls [21]).

### Risk Factors for Severe COVID-19

Risk factors for severe acute COVID-19 illness include older age, male gender, cardiovascular disease, and comorbidities [22]. These factors are associated with a higher risk of hospitalization, ICU admission, and mortality and, therefore, with more severe disease and higher rates of post-COVID ILD. Patients hospitalized for COVID-19 are also more likely to experience persistent symptoms and higher overall symptom burden, most commonly fatigue, dyspnea, sleep disorder, and attention difficulties [23]. Women appear more likely to have persistent symptoms, both respiratory and non-respiratory. This observation may be related to sex-specific differences in immune regulation. Haploinsufficiency of Toll-like receptors 7 and 8 – encoded by loci on the X-chromosome – has been proposed as one mechanism contributing to sex differences in immune responses. This has also been one of several explanations for the female bias in many autoimmune diseases, particularly systemic lupus erythematosus [24]. Notably, systemic lupus erythematosus-related abnormalities have been described in both acute and so-called long COVID-19 [25–27].

### Assessment of Lung Pathologies in Different Types of Sample Specimens

Surgical pathology specimens of the lung can be obtained in many ways, ranging from fine-needle aspirations to needle biopsies, larger forceps and cryobiopsies, wedge resections, segmentectomies, lobectomies, and complete lung resections (explants). As pathology relies on adequate sampling, in general more tissue to be examined is always better, yet this has to be counterbalanced with the feasibility and the expected diagnostic yield contextualized in the potential harm to the patients and the clinical setting [28].

In both neoplastic and non-neoplastic surgical pathology, selection of the method applied needs to be first correlated with the localization of the lesion. Fine-needle aspirations and biopsies yielding the least available tissue to be examined in general are rather useful in preanalytical suspicion of neoplastic diseases, which are also often centrally located, and are not recommended in ILD in general [14]. The larger cryobiopsies and wedge resections are the preferred method for evaluation of ILD [14]; cryobiopsies are useful for central peribronchial lesions and are regarded as very safe procedure in the post-COVID setting [15] wedge resections for lesions in the periphery and of subpleurally located findings that are not assessable by cryobiopsy procedures [16] (see also Table 2). Importantly and what has been shown for ILD in general [17], the diagnostic yield of cyrobiopsies can be increased by centralized multidisciplinary discussion and approximate that of surgical lung biopsies, so it seems to be applicable to post-COVID settings [18]. Segmentectomies, lobectomies, and pneumonectomies are strictly therapeutic procedures carrying unacceptably high risks and should not be performed for diagnostic purposes in order to preserve as much patient lung tissue as possible. Yet, such specimens might be present in the wake of inflammatory complications needing surgical intervention (e.g., lung abscesses), concomitant neoplastic resections, or organ explants in the setting of lung transplantation [29].

**Table 2.** Differential diagnosis of post-COVID fibrotic patterns ([14–19] and own observations)

Pattern	Distribution and potential biopsy method	Architectural distortion	Histopathology
Organizing DAD	Mid-lung zones (cryobiopsy, wedge resection)	Initial injury sites	Myofibroblast proliferation in the alveolar septa, fibroblastic plugs within the distal airspaces
Post-COVID pulmonary fibrosis	Mid-lung zones (cryobiopsy, wedge resection)	Initial injury sites with extension into the adjacent parenchyma	NSIP-like with intussusceptive neoangiogenesis and diffuse thickening of the alveolar walls due to uniform collagen accumulation with mild lympho-histiocytic inflammation
Exacerbation of preexisting ILD	Predominantly basal and subpleural (wedge resection)	Synchronous but spatially and temporally heterogenous lesions	Concurrent but microscopically and respecting the natural disease history heterogenous lesions with subpleurally and paraseptally enhanced fibrosis, fibroblastic foci, microvascular distortion, and honeycombing

ARDS, acute respiratory distress syndrome, COVID, coronavirus disease, DAD, diffuse alveolar damage, ILD, interstitial lung disease.

Along these lines, there are some autopsy studies on COVID-19 patients, which could demonstrate that CT- or ultrasound-guided biopsies are not inferior to classical autopsy asservates of whole organs [30, 31]. However, as a caveat, most of these autopsies were performed in the setting of acute COVID-19 with diffuse lung parenchyma involvement. In cases with only focal lung involvement (central versus peripheral as detected in imaging studies), careful selection of the biopsy method taking into consideration the radiologic presentation, the expected yield, and risks, is crucial to obtain a representative tissue sample for correct diagnosis.

## Mechanisms/Pathophysiology

SARS-CoV-2 infection causes acute lung injury (ALI), and COVID-19 induces hyperinflammatory, angiopathic, and immunothrombotic tissue responses. Understanding their underlying pathophysiology is of paramount importance to understand post-COVID pulmonary sequelae, given the burden of patients with moderate-to-severe COVID-19 who do not recover from ALI or remain at increased risk of developing post-acute lung impairment [32]. Current knowledge on the pathophysiology underlying the development of SARS-CoV-2-associated ALI and post-COVID ILD is summarized in Figure 1 and Table 3, with representative histopathological features shown in Figure 2 and summed up in Table 2.

**Fig. 1.**
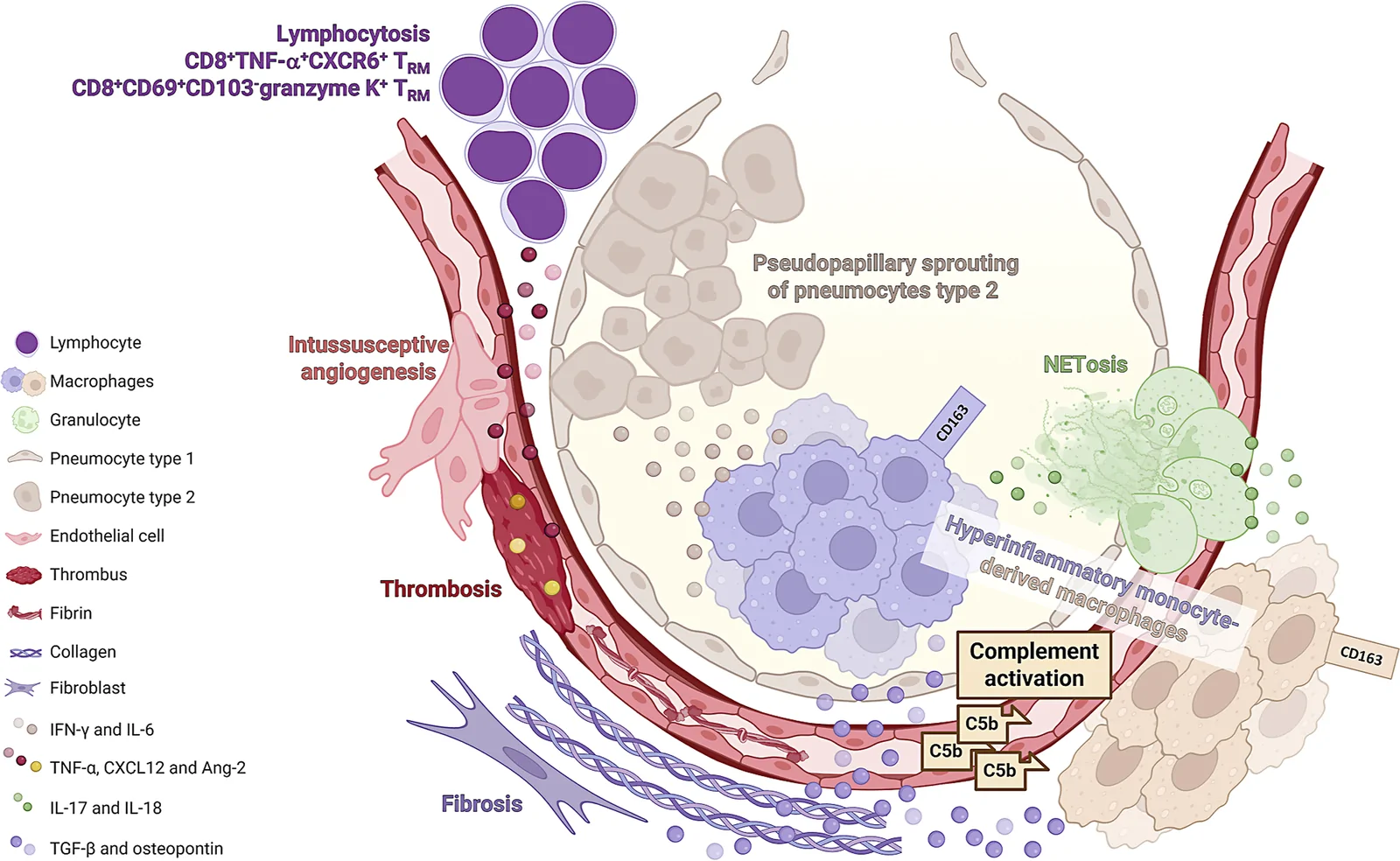
Pathophysiology. Overview of pathological processes in post-COVID ILD, from the perspective of the alveolar unit, according to the current knowledge and linking SARS-CoV-2-induced ALI to PCPF. Pathologic processes are listed clockwise around the alveolus. For clarity, the putative mediators and involved genes are not listed in the scheme. ALI of COVID-19 starts with deregulation of the angiotensin/bradykinin signaling [33] with increased microvascular permeability and endothelial damage and continues with release of the pro-inflammatory and fibrogenic chemokines IFN-γ, IL-1β, IL-6, TNF-α, and TGF-β, mainly from hyperplastic pneumocytes type 2 and inflammatory macrophages. Coagulation factor VIII, vWF, and Ang-2 are released form the damaged endothelial cells as well as they upregulate the *SERPINF1* and *SERPINE1* genes, which encode α2-antiplasmin and PAI-1 that altogether promote microthrombosis on the one hand [34, 35] and contribute to fibroproliferative responses on the other [36]. The above vascular damage and pro-inflammatory cytokine response of ALI are decisive for the ingress and priming of specific lymphocyte and macrophage subsets that deliberate cytokines known to induce marked intussusceptive angiogenesis (e.g., TNF-α and CXCL12), which is otherwise seen in NSIP, promote fibroblast activation (e.g., TNF-α-producing/granzyme K-containing CD8+ TRM lymphocytes) [37, 38], or (hyperinflammatory monocyte-derived M2 macrophages) directly overexpress genes of profibrotic responses (e.g., *SPP1*, encoding osteopontin, which stimulates collagen I production in fibroblasts, and *TGFB1*, encoding TGF-β, the master regulator of wound healing and organ fibrosis) [39]. Complement-mediated microangiopathy is implicated in SARS-CoV-associated ALI [40], and complement dysregulation is enriched in these acute phase patients who still suffer from symptoms after 6 months [41] with highest levels of soluble C5bC6, i.e., membrane attack complexes, that can propagate tissue damage. The strong chemotactic function of the complement and damaged vasculature on neutrophils is also linked to persistent neutrophil-associated immune signature and formation of NETs that further perpetuate local injury and are observable in PCPF [42]. PCPF, post-COVID-19 pulmonary fibrosis; Ang-2, angiopoietin 2; PAI-1, plasminogen activator inhibitor-1.

**Table 3.** Molecular drivers and potential therapeutic targets of post-COVID pulmonary fibrosis (extracted and adapted from references cited in the section “Mechanisms/Pathophysiology”)

Process	Drivers (alphabetic)	Pathophysiology	Potentially active compounds
Angiogenesis	ACE2 and Ang-2, CXCL12, NRP1, TNF-α, VEGF	Intussusceptive angiogenesis, microcirculatory failure with microthrombosis, and complement activation	Matrix metalloproteinase, mTOR, NOTCH, or receptor tyrosine kinase inhibitors (e.g., nintedanib), faricimab (VEGF-A/Ang-2 bispecific), and anti-VEGF/VEGFR antibodies
Neutrophil activation	C5a and C5b, CXCL1, CXCL8, IL-17, IL-18	NETosis (immunothrombosis), persistent epithelial/endothelial injury	Complement inhibitors, disulfiram, gilteritinib, or hydroxychloroquine
M2 polarization	CCL18, IFN-γ, IL-6, IL-10, NETosis, TGF-β	Secretion of TGF-β and osteopontin, other remodeling promoters	CCR2, CSF1, JAK, HDAC, or MEK inhibitors
Fibrogenesis	CTGF, osteopontin, SMAD3, TGF-β	Production of collagen compounds and osteonectin, myo-/fibroblast differentiation	Receptor tyrosine kinase inhibitors (e.g., nintedanib), SMAD3 phosphorylation inhibition, or TGF-β inhibitors (e.g., pirfenidone)

HDAC, histone deacetylase; MEK, mitogen-activated extracellular signal-related kinase; mTOR, mechanistic target of rapamycin; Ang-2, angiopoietin 2.

**Fig. 2.**
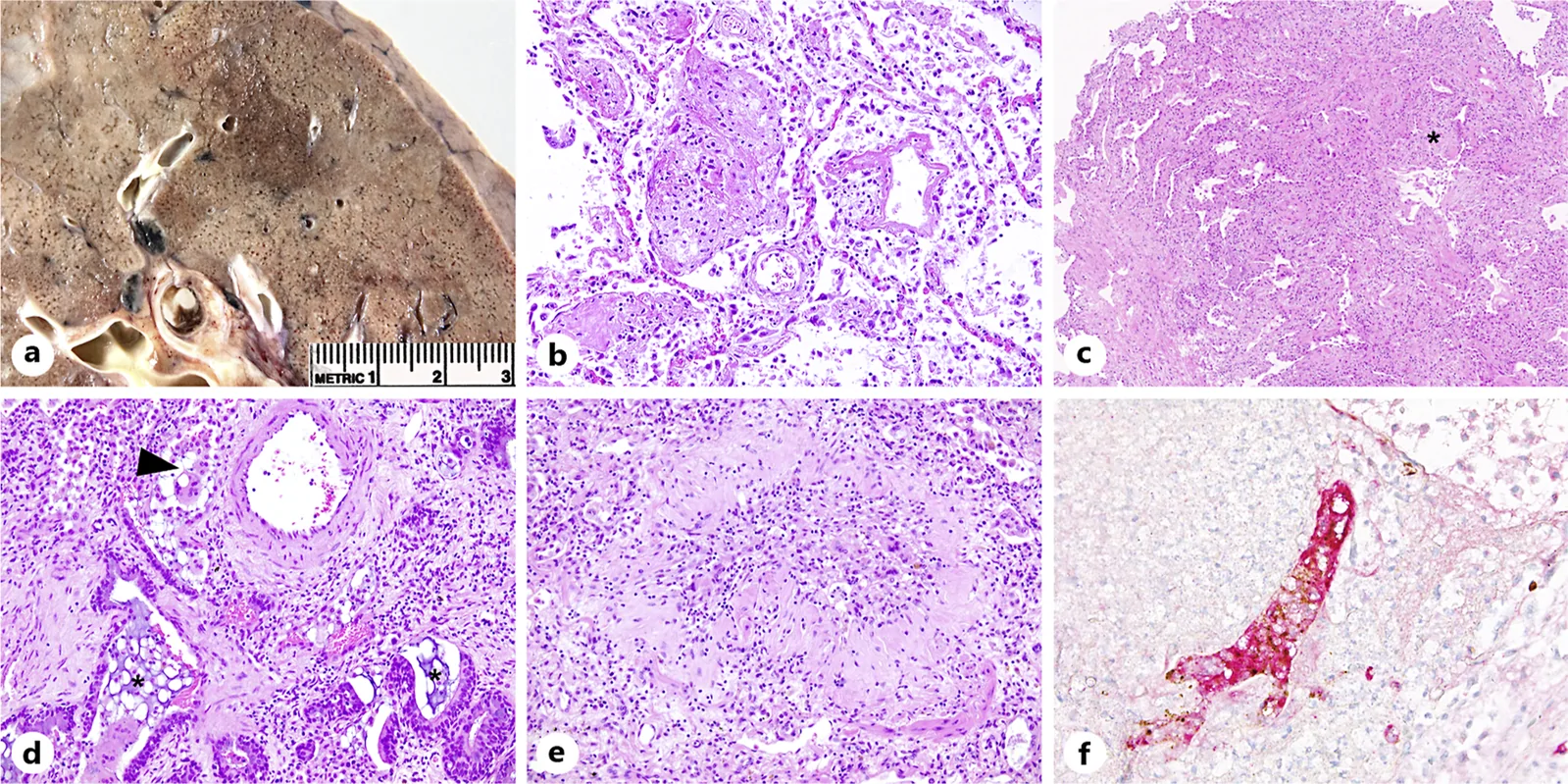
Histopathological features of COVID-associated ALI and ILD. This figure presents histopathological features to be observed in post-COVID-19 lung conditions. **a** Macroscopic image of lung parenchyma of an explanted lung specimen obtained from a 62-year-old male patient without significant comorbidities 185 days after onset of SARS-CoV-2 infection showing condensed lung tissue with focal hemorrhagic areas and a thrombus in the pulmonary artery. **b** Early phase of COVID-19 DAD; H&E. staining of a postmortem specimen obtained from a 75-year-old male patient with a background metabolic syndrome 7 days after onset of infection, 200x. **c** Late organizing stage of COVID-19 DAD with Masson bodies (asterisk); H&E. staining of a cryobiopsy specimen obtained from a 58-year-old female without significant comorbidities 42 days after onset of infection, 200x. **d** Extensive bronchiolocentric interstitial pulmonary fibrosis with bronchiolar/mucinous and transitional cell metaplasia and mucostasis (asterisk) as well as intra-alveolar desquamation of macrophages with occasional multinucleated giant cells (arrowhead); H&E. staining of a wedge resection specimen obtained from a 65-year-old male patient without significant comorbidities 198 days after onset of infection, 200x. **e** Organized thrombus in a peripheral pulmonary artery branch; H&E. staining of an explanted lung specimen obtained 193 days after onset of infection, 100x. **f** Platelet-rich microthrombi in thickened alveolar sepat in a post-acute COVID-19 lung; combined CD42b (brown) and vWF (red) staining of a postmortem specimen obtained from a 71-year-old male patient with a background arterial hypertension 157 days after onset of infection, immunoperoxidase/diaminobenzidine and universal alkaline phosphatase red, 400x.

### Viral Entry and Early Injury

SARS-CoV-2 enters the host cell through the interaction of the angiotensin-converting enzyme 2 (ACE2) with the viral spike (S) protein, supported by the activity of the transmembrane protease serine subtype 2 [43]. This enables SARS-CoV-2 to infect cells that express abundant ACE2: ciliated epithelial cells of the upper respiratory tract, bronchial epithelial cells, type 1 and particularly type 2 pneumocytes [44, 45]. In the early phase of viral pneumonia, the typical DAD pattern with interstitial and intra-alveolar edema and hyaline membranes is not present. The histopathology is dominated by hyperplasia of type 2 pneumocytes with an unusual “patchy” distribution and formation of isolated small aggregates or pseudopapillary sprouts, accumulation of lymphocytes in the interalveolar septa, and dilatation and hyperplasia of the alveolar capillaries [46–48].

Autopsy studies have shown that a subset of patients develops severe viral pneumonia in which lung injury rapidly progresses to DAD, characterized by necrosis of type 1 pneumocytes and endothelial cells, intra-alveolar and interstitial edema, formation of hyaline membranes, microthrombosis of the alveolar capillaries and, after a very short time, myofibroblastic proliferation, hyperplasia of type 2 pneumocytes, and thickening of the intralobular pulmonary artery walls [49]. In surviving patients, this progression has not been well characterized. This progression to a severe form of lung injury appears to be more common in individuals with preexisting chronic, low-grade systemic inflammation (inflammaging) [48]. The development of severe ALI is also promoted by an impaired interferon (IFN) type I response, which is genetically determined or, more frequently, related to the presence of anti-IFN autoantibodies [50, 51]. Closely related to the main viral entry factor, ACE2 and dysregulation of angiotensin-related cascade mechanisms prime the pulmonary microvasculature to fibrogenic remodeling as described in the next paragraph.

### ACE2 and Early Hyperinflammatory Response

During acute disease, cellular infection by SARS-CoV-2 leads to a decrease of ACE2 expression, resulting in reduced activity of the vasoprotective angiotensin 1–7 through the Mas receptor pathway, which contributes to the features of acute COVID-19 DAD. Decreased membranous ACE2 leads to a relative accumulation of angiotensin II, which then binds to angiotensin receptor 1, increasing vascular permeability and neutrophil accumulation. This results in non-cardiogenic pulmonary edema, with leakage of plasma fibrinogen into the interstitium and alveolar spaces, where it polymerizes and forms hyaline membranes. These changes/this process correspond(s) to exudative DAD and SARS-CoV-induced ALI, which clinically manifests as ARDS [47, 52]. DAD paves the way for the first phenotype of fibrotic pulmonary changes after COVID-19, namely, organizing DAD (as a direct consequence of ARDS), characterized by myofibroblastic proliferation, deposition of extracellular matrix, as well as mucinous, transitional cell and squamous metaplasia. In some cases, this process progresses to advanced or even lethal pulmonary fibrosis [53].

Decreased ACE2 also prevents the degradation of des-Arg^9^-bradykinin, which leads to activation of bradykinin receptor 1 [54] and – mainly through unopposed angiotensin II signaling – to the release of the pro-inflammatory and fibrogenic chemokines IFN-γ, IL-1β, IL-6, TNF-α, and TGF-β [55]. Importantly, all these molecules are crucial in the cytokine dysregulation (“cytokine storm”) observed in acute COVID-19. Elevated levels of most of these mediators (except for IFN-γ [56]) seem to be associated with subsequent fibrotic changes [57]. Thus, a substantially altered ACE2 function may, to some extent, be a mechanistic link between acute COVID-19 and certain aspects of post-COVID-19 lung impairment. In addition to ACE2, a key viral entry factor on endothelial cells, neuropilin (NRP) 1, appears to connect vascular to immunopathology in SARS-CoV-2 infection as rendered below.

### Role of NRPs

NRPs are transmembrane proteins functioning as co-receptors for various growth factors and semaphorins. They play a significant role in angiogenesis, neurogenesis, and immune response [58] as well as in the oncogenic setting [59]. NRP1 is predominantly expressed in endothelial cells, vascular smooth muscle cells, epithelial cells of the gastrointestinal and the respiratory tract, and pancreatic beta-cells as well as regulatory T-cells (Tregs). NRP2 is found in smooth muscle cells of the bladder and the intestinal tract and plays a major role in lymphangiogenesis. Regarding SARS-CoV-2 infection, NRP1 could be identified as a key point of entry of the virus into endothelial cells, B- and T-lymphocytes (especially Tregs), and macrophages bypassing the lack of ACE2 receptors on the surface of these cells. In a comprehensive autopsy study, Dette et al. [60] could confirm the direct attachment of NRP1 and NRP2 to SARS-CoV-2 spike fragments S1 and S1’. In situ analyses showed the colocalization of SARS-CoV-2 RNA and NRP1 in cells negative for ACE2.

NRP1 appears to function not only as an alternative (to ACE2) entry point for SARS-CoV-2, but is also involved in inducing neoangiogenesis, vascular endothelial damage, and progressive microangiopathy with thrombosis. In contrast, NRP2 exerts an immunomodulatory effect that may mitigate processes associated with long COVID and pulmonary remodeling [61]. Although the role of NRPs in post-COVID lung disease remains insufficiently characterized, the involvement of NRP1 suggests that a range of immunological and vascular pathologies may be linked to mechanisms related to this alternative viral entry pathway via endothelial, monocytic, and lymphoid cells.

### Vascular Damage in COVID-19

As endothelial cells express ACE2 and especially NRP1, it is not surprising that viral inclusion particles were detected in these cells [34, 62]. Whether SARS-CoV-2 has direct angiopathic effects is still under debate, but the SARS-CoV-2 S and nucleocapsid (N) proteins can deposit on endothelial cells [63], and the S protein can bind to fibrinogen, giving rise to fibrin microclots [64]. Thus, it is not surprising that moderate and severe acute COVID-19 are associated with a hypercoagulable state characterized by thrombotic complications that contribute substantially to morbidity and mortality [47, 65]. Coagulation factor VIII, von Willebrand factor (vWF), and angiopoietin 2 are stored in endothelial cells and released upon endothelial injury [66]. As a reflection of endothelial damage, these molecules are elevated in COVID-19 and have been shown to predict clinical outcome, particularly mortality [67]. There is compelling evidence of persistent prothrombotic diathesis with a 10-fold to 30-fold increased risk of thrombotic complications persisting for up to 1 year after acute COVID-19 [68, 69].

The best evidence for the central role of vascular damage of acute COVID-19, referred to as angiocentricity, comes from radiologic, gross, and microscopic observations. CT imaging of COVID-19 ALI shows patchy, mainly peripherally distributed ground-glass opacities (GGOs) with signs of vasodilatation [70, 71]. The patchy distribution corresponds to a pattern related to the secondary pulmonary lobule, which represent the watersheds of the pulmonary circulation, i.e., the functional circulation over the pulmonary arteries, and bronchial circulation, i.e., the nutritional circulation of the lung over the bronchial arteries [72, 73]. GGO corresponds histopathologically to exudative DAD, and the signs of vasodilatation predominantly reflect capillary dilatation, congestion, and microthrombosis within the alveolar capillary network [34]. Importantly, mosaic attenuations following the secondary lung lobe pattern distribution can also be observed in persistent lung injury, providing a mechanistic link between ALI and post-COVID pulmonary damage [74]. In accordance with the above-mentioned hypercoagulable state, SARS-CoV-2-induced DAD has an almost 10-fold higher microscopic prevalence of alveolar capillary fibrin microthrombi compared with influenza virus-induced DAD, indicating substantially more pronounced local microvascular damage in COVID-19 [34]. In more than one-third of patients who died from COVID-19, thrombi and platelets have been identified within hyaline membranes of the DAD as well as in vWF-rich thrombi in alveolar microvessels [47, 75]. Furthermore, individuals with PASC were found to have increased levels of vWF and fibrin chains, consistent with endothelial damage [35]. In ALI, alveolar capillaries are clearly disrupted with irregularities on the luminal surface, indicating endothelial inflammation and destruction, narrowed lumens indicating microthrombus deposits, and tortuous and dilated bronchial vessels indicating bronchial artery hypertrophy and vascular remodeling [34]. At the ultrastructural level, endothelial cells show swelling, dissolution of intercellular junctions, and detachment from the basement membrane, all hallmarks of severe endotheliopathy. An important aspect linking the acute microthrombotic events of COVID-19 ALI to fibrotic changes is the fact that uncontrolled coagulation generally considerably contributes to fibroproliferative responses [36].

### Transition from Acute to Subacute Disease

#### Role of Complement

The transition from acute to subacute COVID-19 is not fully understood, but is likely driven by molecular mechanisms linked to what has been observed in longitudinal peripheral blood studies showing that interferon-stimulated gene (ISG) expression is elevated in early COVID-19 and declines over time [76], whereas complement activation contributes to lung injury in patients with severe COVID-19 who survive beyond 2 weeks with symptomatic disease [77]. Consistently, depositions of C3d and C5b-9 terminal complement complex (TCC) were observed in lung tissue, indicating complement activation in low expression of ISG patients [78]. Complement-mediated microangiopathy in COVID-19 is now considered important [40], as it has been commonly implicated in SARS-CoV-associated ARDS [79]. In the context of pathway dysregulation in PASC, the complement cascade and alternative complement activation pathways were recently found to be most enriched in the acute phase of the disease in patients still suffering from symptoms after 6 months [41] These patients exhibit the highest levels of soluble C5bC6 during the acute phase of disease, which persist after 6 months, as along with reduced levels of C7-containing TCC (C7 incorporates TCC into cell membranes), suggesting consumption through incorporation of the C7 TCC into damaged tissue. In addition, increased lytic complement activity driven by classical C2 activation, increased levels of the C3 degradation product C3d, and evidence of low-grade hemolysis have been observed in PASC, along with increased levels of vWF and the associated coagulation factor VIII [41]. Moreover, these PASC patients also demonstrated elevated anti-cytomegalovirus and anti-Epstein-Barr virus IgG levels, which correlate with C2 levels, confirming the role of reactivation of herpes family viruses in long COVID, not only as a consequence of immune dysregulation, but also as potential triggers of complement activation [29, 41]. Being highly attractive for neutrophilic granulocytes, the cumulating complement compounds in patients surviving acute COVID-19 pave the way toward a neutrophil-related immunopathology as described next.

#### Role of Neutrophil Extracellular Traps

Whether complement activation-related tissue damage has a role in impaired regeneration remains to be elucidated. However, given the strong chemotactic function of C5a on neutrophils in COVID-19 ALI, complement activation is likely linked to another aspect of chronic lung injury, namely, a persistent neutrophil-associated immune signature and the closely associated formation of neutrophil extracellular traps (NETs) [80]. NETs are networks of extracellular fibers composed primarily of neutrophil-derived DNA with a high potential for host tissue damage, which is termed NETosis [81, 82]. Indeed, individuals with persistent interstitial lung abnormalities after COVID-19 exhibit elevated levels of IL-17C, which is crucial in neutrophil recruitment, and an enriched neutrophil chemotaxis pathway [83]. Elevated levels of the neutrophil-attracting chemokines CXCL1 and CXCL8 (IL-8) correlate negatively with lung function tests, and the inflammasome-associated cytokine IL-18 and its receptor correlated positively with the radiological disease extent on CT scans, suggesting that a persistent neutrophil-associated pro-inflammatory signature is associated with post-COVID ILD [42]. In addition, affected patients have higher neutrophil counts, increased plasma levels of myeloperoxidase, and H3R8-citrullinated nucleosomes, suggesting a persistent neutrophil signature and NETosis, both of which are associated with and predictive of severe and fatal acute COVID-19 [35, 84]. Interestingly, experimental in vitro administration of NETs to A549 cells (adenocarcinoma-derived human alveolar basal epithelial cells) induces gene expression changes consistent with epithelial-mesenchymal transition with increased *ACTA2* and reduced *CDH1* levels, similarly to what is observed in epithelial-mesenchymal transition in general and in fibrotic remodeling in particular, linking NETosis to the development of post-COVID ILD [42]. The relevance of this “neutrophil signature” is further supported by the fact that complete healing in the few patients after 12 months of observation was associated with a decrease in IL-17C, CXCL8, myeloperoxidase, and H3R8 citrullinated nucleosomes [42]. In addition to direct tissue damage and immunothrombosis by NETosis, the latter seems to exert effects on macrophages, driving their M2 polarization to tissue remodeling cells [85] that seem to play a decisive role in post-COVID ILD by fueling the TGF-β pathway as summarized below.

#### Role of Macrophages

Lung-resident macrophages and those recruited from the circulation have an essential role in the onset, development, and termination of ARDS, compared with other myeloid cells [86]. Consistent with this and compared to bronchoalveolar lavage (BAL) from healthy individuals, BALs from patients with severe COVID-19 contain a higher proportion of chronic hyperinflammatory monocyte-derived macrophages and a lower proportion of anti-inflammatory and antigen-presenting alveolar macrophages indicating possible pathologic macrophage activation. Alongside, in the peripheral blood of patients with mild COVID-19, an increase in HLA-DR^hi^CD11c^hi^ inflammatory monocytes with an ISG signature and an increase in dysfunctional HLA-DR^lo^CD11c^lo^CD69^+^CD226^+^ monocytes can be observed [37]. Activation of monocytes that triggers an inflammatory lung response, mainly by upregulation of type I IFN in severe and type III IFN in moderate disease and ISG, which is quite pronounced in early disease with high viral load, can be achieved by several mechanisms. It may be either the result of direct infection of monocytes by SARS-CoV-2 or indirect infection by engulfing IgG antibody-coated viruses via their Fcγ receptors, or be the sum effect of the pathogen-associated molecular pattern response of pattern recognition receptors including Toll-like receptors 3, 4, 7, and 8 [87]. It seems that the former hyperinflammatory monocyte-derived lung macrophages appear to be instrumental in a deleterious loop of pro-inflammatory and pro-fibrotic cytokine release and effector cell recruitment in COVID-19. This amplifies tissue damage at the site of infection and likely contributes to the development of post-COVID ILD [88]. Indeed, SARS-CoV-2-exposed monocyte-derived macrophages, designated CD163/legumain-Mφ, show an enrichment of fibrosis-associated gene signatures with overexpression of, e.g., *SPP1* (encoding osteopontin, which stimulates collagen I production in fibroblasts)*, TGFB1* (encoding the master regulator of wound healing and organ fibrosis, *TGF-β*) as well as genes encoding scavenger receptors and proteins involved in recognition of tissue damage and/or wound healing and profibrotic responses. Notably, these macrophage populations share significant similarities with those observed in IPF, establishing another mechanistic link between acute COVID-19 and post-COVID ILD [39]. The overexpression of these profibrotic molecules may be mechanistically linked to the increased numbers of pulmonary fibroblasts that produce collagen triple helix repeat containing 1 protein (CTHRC1) and secreted protein acidic and cysteine rich (SPARC, also called osteonectin) [89], and may be decisive for the development of fibrotic-like lung lesions observed in post-COVID-19 patients suffering from persistent respiratory sequelae.

#### Role of Lymphocytes

Severe acute COVID-19 is characterized by lymphopenia [90], which resolves rapidly after recovery [91] and is therefore likely attributable to sequestration of circulating lymphocytes in the lungs. Interestingly, lymphopenia seems to predict PASC, particularly post-COVID ILD [92]. In BAL fluid from patients with acute COVID-19, lymphocytes are predominantly T cells and reflect disease severity [93]. In mild COVID-19, CD8+ T-resident memory (TRM) and CD4+ T-helper-17 cells predominate, undergo active clonal expansion, and are characterized by good effector functions, whereas in critical COVID-19, levels of T cells – especially CD8+ T cells – are low, and these cells exhibit a naïve phenotype [37]. In post-COVID lung disease, dysregulated immune responses, in particular exaggerated responses of certain subsets of airway CD8+ T cells, have been observed [38]. Convalescent patients show adaptive changes in the lung lymphoid compartment and functional immunologic memory with probably protective effects [94], but patients with impaired lung function show a dysregulated CD8+ T-cell compartment with increased numbers of CD8^+^CD69^+^CD103^-^ T_RM_ cells that express high levels of granzyme K [37] and may promote fibroblast activation [38, 95]. In addition, a specific CXCR6^hi^ and TNF-α overexpressing population of pathogenic, tissue-resident CD8+ T cells was identified, which negatively affects lung gas exchange function and has been associated with the radiographic presence of reticular consolidations and GGOs [38]. This T-cell population is similar to a population that drives tissue damage in nonalcoholic steatohepatitis, another chronic inflammatory and fibrosing disease [96].^.^Importantly, the CD8^+^CD69^+^CD103^-^ T_RM_ cells also exhibit a gene expression profile consistent with tonic (i.e., low but constitutive) T cell-receptor signaling triggered by either persistent SARS-CoV-2 or autoantigens, as has been suggested for humoral immune responses [97]. TNF-α overexpression by these T cells may further contribute to post-COVID ILD and fibrotic remodeling, given the established role of TNF-α in pulmonary fibrosis [98].

In addition, the role of CD4^+^ Tregs with a primarily immunosuppressive function is increasingly recognized [99]. These cells might help prevent the hyperinflammatory setting of long COVID. This T-cell subgroup expresses NRP1, which might explain the dysfunction of the immune system as the SARS-CoV-2-infected Tregs are eliminated by the virus and their impact on immune homeostasis is lost [100].

### Features of Post-COVID Pulmonary Fibrosis

The observation of intussusceptive angiogenesis within the alveolar compartment provides a mechanistic link between COVID-19 ALI and the second phenotype of fibrotic changes in post-acute COVID-19 lungs, since angiogenesis is considered a key contributor to the development of pulmonary fibrosis as explained below [18, 34, 53, 101]. The characteristic feature of post-COVID ILD is the formation of intussusceptive endothelial columns that span the lumen of small vessels and create two vessels from the former vessel by longitudinal expansion. It is considered a very efficient microvascular expansion with minimal requirement for energy and endothelial proliferation [102]. Indeed, at the single-cell level, the transcriptomes of lung endothelial cells of patients who died of COVID-19, particularly in the late acute phase, largely resemble those of IPF: increased extracellular matrix deposition and/or remodeling and altered immunomodulation, but with enriched expression of genes involved in cellular stress, attenuated immunomodulation, and impaired barrier function, and reduced maintenance of endothelial integrity [103]. Post-COVID ILD strongly resembles NSIP/organizing pneumonia and to a lesser extent usual interstitial pneumonia (UIP) [18, 29, 34, 53, 101]. In NSIP, vascular remodeling is characterized by marked intussusceptive neoangiogenesis, in contrast to UIP or IPF, in which sprouting neoangiogenesis into the fibrotic areas is characteristic [34]. Consistently, post-COVID ILD shows a greater overlap of gene expression with NSIP than with UIP in general and UIP in the setting of IPF, but also some distinctive features compared to NSIP, such as the overexpression of *FGF2*, *SERPINF1*, and *NRXN1*. This could be of therapeutic importance, as NSIP/organizing pneumonia respond particularly well to corticosteroids and other immunosuppressive therapies [104].

At the molecular level, damaged endothelial cells lose their ability to maintain physiological functions, in particular their antithrombotic activity at the luminal surface (e.g., decreased expression of CD39, prostaglandin 2, tissue factor pathway inhibitor) as well as reduced production of tissue plasminogen activator and nitric oxide [105]. Moreover, the endothelium not only loses its antithrombotic properties but also exerts an opposite set of functions by actively producing thromboxane, antiplasmin, and plasminogen activator inhibitor-1 [106]. In fact, the *SERPINF1* and *SERPINE1* genes, which encode α2-antiplasmin and plasminogen activator inhibitor-1, are among the most upregulated genes in patients who succumb to COVID-19 [34]. *SERPING1*, which encodes the C1 esterase inhibitor, is upregulated in acute COVID-19, and *C1QA, C1QB, C2,* and *C3*, which encode the corresponding complement compounds, are upregulated in late COVID-19 [78]. Indeed, deregulation of the complement cascade has been identified as part of a distinct immunopathologic signature of a specific subgroup of acute COVID-19 patients who remained hospitalized for ≥10 days, subsequently died, and had more profound lung damage, low expression of ISG, low viral loads, and abundant infiltrating-activated CD8+ T cells and macrophages [78]. In this context, NRPs may play a crucial role, particularly in light of emerging therapeutic approaches targeting these pathways in pulmonary fibrosis [61].

Taken together, acute SARS-CoV-2-induced disruption of angiotensin-related signaling pathways, development and organization of DAD, alveolar and peribronchial thrombotic disease, intussusceptive angiogenesis, and deregulation of complement functions establish a link between COVID-19 ALI and its progression to chronic lung injury. Overall, chronic lung damage after SARS-CoV-2 infection appears to be a complex tissue response in which multiple features of COVID-19 ALI evolve into a chronic phase characterized by endotheliopathy, angiopathy, thrombotic diathesis, development of DAD, continuous tissue damage due to complement dysregulation, persistent neutrophil- and T_RM_-driven damage, autoimmune phenomena, and profibrotic macrophage signaling. Understanding these mechanisms provides a critical foundation for exploring specific therapeutic interventions in patients with PASC, particularly those with post-COVID lung injury.

Ongoing research in the field will help better understand prevalence of dyspnea and injury mechanisms, as well as the most efficacious diagnostic, interventional, and preventive strategies for the post-COVID-19 patient cohort, thereby advancing patient care, especially considering the non-responsiveness to anti-fibrotic therapy approaches observed in the few larger available trials so far [107, 108]. Nintedanib, which has been successfully applied in cases of progressive ILD, has not shown the expected efficacy in post-COVID-19 patients. Similar results were observed using pirfenidone. However, both clinical trials showed an overall improvement of respiratory function in both the study and the placebo group indicating that the overall risk of disease progression is lower and the possibility of improving lung function is higher in post-COVID-19 patients than other ILD-patients. This could hypothetically be explained by the different pathomechanisms involved in “classical” ILD and post-COVID lung injury such as the focus on vascular remodeling in post-COVID lung injury and a higher tendency toward ongoing fibrotic remodeling in other ILD.

## Outlook

The effects of SARS-CoV-2 infection on the respiratory system remain an active area of investigation. Considerable progress has been made in understanding the PASC, as well as the contribution of acute viral infections in the development of ILD. Ongoing research includes a deeper characterization of the pathophysiology underlying persistent clinical symptoms, injury resolution and repair mechanisms, and a potential acquired susceptibility for subsequent complications.

Persistent respiratory symptoms, notably dyspnea and pathological breathing patterns, are frequently observed in those recovering from COVID-19. Both pulmonary and extrapulmonary factors, including diminished exercise capacity, may lead to enduring and debilitating breathlessness in individuals affected by post-COVID-19 syndrome. After severe COVID-19, objectifiable diagnoses of chronic lung injury include post-COVID ILD, together with airway and pulmonary vascular abnormalities, necessitating thorough clinical evaluation with specialized diagnostic procedures and tailored therapeutic regimens. Further long-term exploration is required to elucidate the precise nature of pulmonary and extrapulmonary perturbations that result in dyspnea, including longitudinal prospective studies to delineate long-term complications more precisely. Ongoing research in the field will help better understand prevalence of dyspnea and injury mechanisms, as well as the most efficacious diagnostic, interventional, and preventive strategies for the post-COVID-19 patient cohort, thereby advancing patient care.

Thus, further research priorities should focus on the differentiation between pulmonary and extrapulmonary contributions to respiratory distress and reduced quality of life. From a pulmonary perspective, a comprehensive understanding of all compartments of the lung is needed. This issue may be exemplified by the fact that the mechanisms underlying new onset or exacerbated airway disease (such as asthma or COPD) following a SARS-CoV-2 infection remain unclear, despite being common in clinical practice. Furthermore, treatment strategies for acute SARS infection that minimize or prevent the evolution to persisting pulmonary injury should be further improved. In particular, the role of an add-on therapy with antifibrotic agents in severe acute disease remains unclear. As there have been few severe cases since the end of the pandemic, this question remains unresolved for now. However, it remains highly relevant in the context of potential future SARS-CoV-2 outbreaks or similar viral pandemics.

## Conflict of Interest Statement

Thomas Menter and Alexandar Tzankov were members of the journal’s Editorial Board at the time of submission. Kirsten D. Mertz has no conflict of interest to declare.

## Funding Sources

The authors were supported by the Basel (formerly Botnar) Research Centre for Child Health.

## Author Contributions

TM, MK, and AT conceived and wrote the paper. AT designed the figures.
